# A perspective on precision therapeutics in polycystic ovary syndrome: integrating multi-omics, AI-driven stratification, and emerging biologics

**DOI:** 10.3389/fendo.2026.1787902

**Published:** 2026-03-24

**Authors:** Philippe Pinton

**Affiliations:** Health & Life Sciences, Shiroito Co. Ltd, Tokyo, Japan

**Keywords:** AMH, artificial intelligence, biologics, disease-modifying-therapies, multi-omics, patient stratification, polycystic ovary syndrome, precision medicine

## Abstract

Polycystic ovary syndrome (PCOS) is one of the most prevalent and heterogeneous disorders in reproductive endocrinology, contributing to substantial reproductive, metabolic, and psychological morbidity. Despite major advances in genomics, neuroendocrinology, and systems biology, translation into disease-modifying therapies remains limited. This Perspective argues for a strategic shift toward precision medicine, supported by molecular subtyping, multi-omics profiling, and artificial intelligence (AI). These tools now enable biologically informed patient stratification and the identification of novel therapeutic targets. We highlight emerging strategies - including anti-Müllerian hormone (AMH) neutralization, neuroendocrine modulators, metabolic agents, and next-generation biologics - and discuss the regulatory, ethical, and operational considerations required to accelerate innovation. A coordinated, multi-disciplinary approach integrating computational analytics, biomarker-driven endpoints, and patient-centered outcomes is essential to close the longstanding gap between scientific potential and clinical reality.

## 1 Introduction

Polycystic ovary syndrome (PCOS) is the most common endocrine disorder among women of reproductive age, affecting 6–13% globally (1, 2). Its clinical manifestations span reproductive, metabolic, dermatologic, cardiovascular, and psychological domains (3). The economic burden is substantial, with healthcare costs and productivity losses reaching billions annually (4). Despite this impact, PCOS remains primarily managed through symptomatic treatments, with no approved therapies targeting the underlying biological mechanisms (5).

Recent advances in genomics, neuroendocrinology, and systems biology have revealed distinct molecular subtypes and complex interactions between metabolic, reproductive, and environmental factors (6, 7). Yet translation of these insights into disease-modifying therapies has lagged. Persistent challenges - including heterogeneous diagnostic criteria, lack of validated biomarkers, and limited integration of patient-reported outcomes - continue to hinder progress (8, 9). PCOS thus represents both a major unmet medical need and a unique opportunity to redefine innovation in women’s health through precision medicine, AI-enabled stratification, and emerging biologics (10).

## 2 Perspective

**Figure 1:**
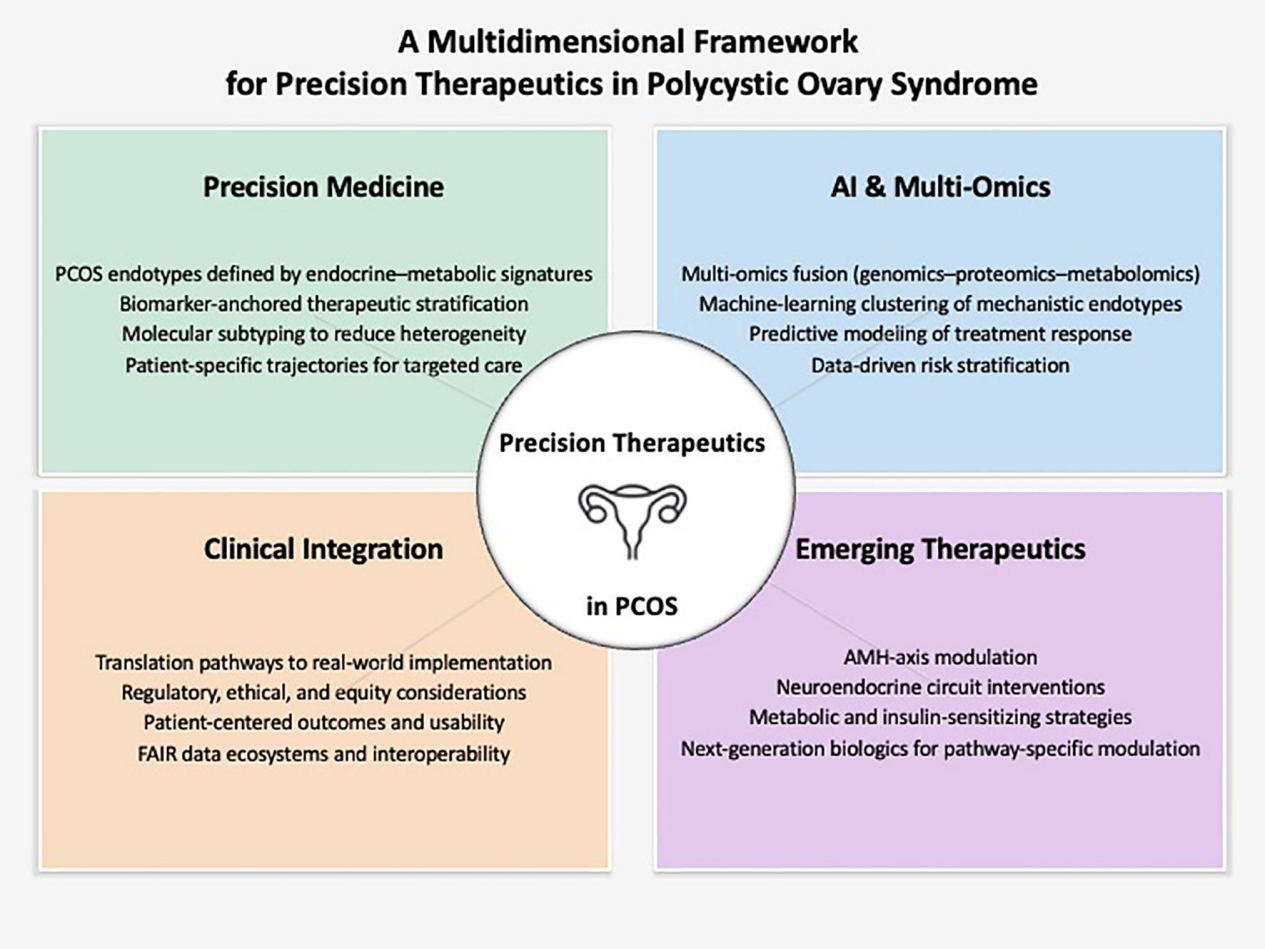
Conceptual framework for precision therapeutics and AI-driven innovation in polycystic ovary syndrome (PCOS). This infographic illustrates four interconnected domains shaping the future of PCOS research and therapeutic development. (1) Precision Medicine -molecular subtyping, biomarker-guided stratification, and individualized endocrine–metabolic profiling. (2) AI and Multi-Omics - integration of computational analytics with genomic, hormonal, metabolic, and imaging data to refine patient stratification and predict treatment response. (3) Emerging Therapeutic Strategies - AMH-axis neutralization, neuroendocrine modulators, metabolic agents, and next-generation biologics targeting pathway-specific mechanisms. (4) Clinical Integration - operational, regulatory, ethical, and patient-centered considerations required to translate scientific advances into disease-modifying therapies.

PCOS research is at an inflection point: biological understanding has advanced rapidly, yet clinical translation remains slow and fragmented. We argue that the persistent gap between mechanistic insight and therapeutic innovation reflects structural limitations in how PCOS is defined, studied, and managed. Integrating precision medicine, multi-omics stratification, and AI-enabled analytics may offer one of the most promising routes to disease-modifying therapies. By synthesizing emerging scientific advances and identifying actionable opportunities, this Perspective aims to catalyze a shift toward more targeted, biologically grounded, and patient-centered approaches to PCOS care. **Figure 1** presents the conceptual framework proposed in this Perspective, integrating precision medicine, multi-omics analytics, emerging therapeutic strategies, and clinical implementation pathways.

## 3 Progress and persistent gaps in PCOS research

Large-scale genomic and multi-omics studies have identified pathways involved in androgen excess, insulin resistance, and ovarian dysfunction. These insights have revealed biologically distinct PCOS subtypes, including metabolic-dominant and neuroendocrine-dominant phenotypes (11, 12). Despite this progress, translation into disease-modifying therapies remains limited. Most available treatments target symptoms -such as menstrual irregularity, infertility, or metabolic risk - rather than underlying mechanisms. Persistent challenges include the absence of validated biomarkers, lack of consensus diagnostic criteria, and limited integration of patient-reported outcomes. These gaps contribute to delayed diagnosis, fragmented care, and persistent unmet needs.

## 4 Rethinking drug development in PCOS

Traditional drug development has been hindered by PCOS heterogeneity, reliance on short-term symptom-based endpoints, and insufficient patient stratification (13). Several promising therapeutic candidates have failed in clinical trials due to inadequate biomarker integration and endpoints misaligned with patient priorities (14). Advances in molecular profiling, real-world data analytics, and AI now enable more precise identification of PCOS subtypes. Integrating multi-omics biomarkers, patient-reported outcomes, and adaptive trial designs may improve clinical relevance and success rates (15). Collaborative efforts among academia, industry, and regulatory agencies are essential to establish meaningful endpoints and accelerate translation of basic science into clinical innovation (16).

## 5 Precision medicine and emerging therapeutic targets

Precision medicine aims to match therapies to the underlying biology of distinct PCOS subgroups. Among emerging targets, anti-Müllerian hormone (AMH) has gained particular attention. AMH plays a central role in folliculogenesis and is markedly elevated in PCOS (6, 12). Neutralizing AMH with monoclonal antibodies represents a novel therapeutic strategy with potential to restore ovulatory function (17). This approach remains at the preclinical stage, with proof-of-concept data emerging primarily from animal models.

Beyond AMH, several therapeutic classes are under investigation (18–21).

### 5.1 Metabolic pathway modulators

- GLP-1 receptor agonists (e.g., liraglutide, semaglutide) improve weight, insulin sensitivity, and menstrual regularity. These agents are supported by multiple early-phase and mid-phase clinical trials in individuals with PCOS.
- SGLT2 inhibitors (e.g., empagliflozin) show promise for metabolic phenotypes. Evidence remains preliminary, with current data derived from small pilot studies.

### 5.2 Neuroendocrine modulators

- Kisspeptin agonists and.
- Neurokinin-3 receptor antagonists aim to normalize GnRH neuron activity and LH pulsatility, potentially restoring ovulation. Clinical development is ongoing, with early proof-of-concept trials demonstrating normalization of LH pulsatility.

### 5.3 Steroidogenesis inhibitors

Targeting CYP17A1 or CYP11A1 offers a direct approach to reducing androgen excess. These compounds are currently in early-stage clinical evaluation for PCOS-related hyperandrogenism.

### 5.4 Epigenetic and gene-based therapies

Epigenetic modifiers and gene therapy approaches are being explored to reverse PCOS-related gene expression changes. These strategies remain entirely preclinical, with ongoing efforts focused on mechanistic validation and delivery feasibility.

### 5.5 Circadian and melatonin pathways

Melatonin receptor modulators may help restore circadian regulation of ovarian function.

### 5.6 Nutraceuticals

Inositols and berberine continue to show benefits for insulin sensitivity and ovulatory function.

### 5.7 Novel biologics

Anti-androgen antibodies and gonadotropin modulators represent emerging biologic strategies with potential to address multiple pathophysiological pathways. Most biologic candidates are in exploratory or preclinical development, with translational pathways still being defined.

## 6 AI-driven patient stratification

AI offers transformative potential for PCOS classification and therapy optimization. Traditional diagnostic systems rely on clinical criteria that fail to capture biological heterogeneity. Machine learning models can integrate multi-omics data, hormonal profiles, imaging, and real-world health records to identify distinct PCOS subtypes with unique pathophysiological drivers (22–25). Clustering approaches have revealed metabolic-dominant versus neuroendocrine-dominant phenotypes, which may guide selection of GLP-1 receptor agonists or neurokinin antagonists. Future AI-powered decision-support tools embedded in electronic health systems could provide real-time stratification and personalized treatment recommendations (26). Despite these advances, external validation across diverse populations, model generalizability, and regulatory requirements for transparency and explainability remain key challenges for clinical implementation.

## 7 Newest insights in PCOS research

Recent advances in computational and molecular medicine are reshaping the landscape of PCOS research. Federated learning approaches now enable multi-center AI model training without requiring direct data sharing, addressing privacy concerns while improving model generalizability across diverse populations. Explainable AI has emerged as a critical requirement for clinical adoption, ensuring transparency in diagnostic and therapeutic decision-support systems. Multi-omics profiling - including genomics, transcriptomics, proteomics, and metabolomics - is increasingly used to identify biologically meaningful PCOS subtypes and guide personalized therapy. Combination therapies, such as GLP-1 receptor agonists paired with SGLT2 inhibitors, may demonstrate synergistic benefits for metabolic phenotypes. In parallel, gene editing and RNA-based therapeutic platforms are being explored as future avenues for disease-modification. Collectively, these developments highlight a rapidly evolving field where computational and molecular tools converge to refine PCOS classification and therapeutic targeting.

## 8 The transformative potential of AI in clinical care

AI-based tools are poised to transform clinical care in reproductive endocrinology. Machine learning models integrating electronic health records, hormonal profiles, and ultrasound imaging have achieved high diagnostic accuracy for PCOS, with some deep learning systems exceeding 95% accuracy in specific datasets (27). AI-driven clustering approaches have identified novel PCOS subtypes with distinct metabolic and neuroendocrine signatures, while predictive modeling enhances risk assessment for long-term complications such as type 2 diabetes and cardiovascular disease (28). Regulatory frameworks increasingly emphasize transparency, bias mitigation, and patient data privacy, supporting the responsible integration of AI into clinical workflows (29). Ensuring reproducibility, mitigating algorithmic bias, and establishing robust post-deployment monitoring frameworks will be essential for safe and equitable adoption. As these tools mature, AI-enabled decision support may provide real-time stratification, individualized treatment recommendations, and continuous monitoring, ultimately improving outcomes and reducing diagnostic delays (30).

## 9 Environmental and epigenetic factors

Environmental exposures, including endocrine-disrupting chemicals (EDCs), have been increasingly implicated in PCOS risk and phenotype expression (31). Lifestyle factors such as diet, physical activity, and sleep patterns modulate metabolic and hormonal pathways central to PCOS pathophysiology. The gut microbiome has emerged as a key mediator linking environmental inputs to metabolic and reproductive outcomes, with alterations in microbial composition and barrier function observed in individuals with PCOS (32). Epigenetic mechanisms - including DNA methylation, histone modifications, and non-coding RNAs - may contribute to both individual susceptibility and potential transgenerational transmission of PCOS traits. Recent mechanistic studies have further detailed the epigenetic pathways implicated in PCOS (33). Interventions targeting the microbiome, epigenetic regulators, or lifestyle behaviors are under active investigation and may complement pharmacological therapies (34). Understanding these environmental and epigenetic influences is essential for developing holistic, personalized treatment strategies.

## 10 Case study: AMH as a therapeutic target

Anti-Müllerian hormone (AMH) levels are typically two- to four-fold higher in individuals with PCOS (6). Elevated AMH disrupts folliculogenesis, amplifies LH pulsatility, and contributes to androgen excess (12). Preclinical studies demonstrate that neutralizing AMH with monoclonal antibodies can restore ovulatory cycles and normalize androgen production (13). However, challenges remain, including potential immunogenicity, manufacturing complexity, and cost considerations. Advances in patient stratification using AMH levels, multi-omics markers, and AI-based clustering may help identify subgroups most likely to benefit from AMH-targeted therapies. This case study illustrates how integrating molecular insights with precision therapeutics can open new avenues for disease-modifying interventions in PCOS.

## 11 Patient-reported outcomes and quality of life in PCOS trials

Patient-reported outcomes (PROs) and quality-of-life (QoL) measures are increasingly recognized as essential components of PCOS research and clinical trials. Traditional endpoints - such as ovulation rates, androgen levels, or metabolic markers - capture important physiological changes but may not fully reflect the lived experience of individuals with PCOS. Validated PRO instruments, including the Polycystic Ovary Syndrome Health-Related Quality of Life Questionnaire (PCOSQ), assess domains such as hirsutism, menstrual irregularity, emotional distress, and fertility concerns (35). Recent guidelines advocate for the routine inclusion of PROs and QoL metrics in both interventional and observational studies (36). Digital PRO tools and real-world data platforms enable long-term monitoring beyond the clinical setting, supporting more patient-centered evaluation of therapeutic benefit. Incorporating PROs into regulatory and payer discussions may strengthen the case for emerging therapies by demonstrating meaningful improvements in daily functioning and well-being.

## 12 Overcoming systemic barriers

Despite scientific progress, innovation in PCOS research and care remains constrained by systemic barriers. Fragmented care pathways, limited funding for women’s health, regulatory uncertainty, and payer resistance have historically slowed the development and adoption of new therapies. Recognizing PCOS as a chronic, high-burden condition is essential to justify dedicated resources and integrated management strategies.

Harmonizing diagnostic criteria, developing unified coding systems, and implementing value-based reimbursement models could incentivize innovation and improve access to emerging treatments. Multi-disciplinary collaboration - bringing together endocrinologists, gynecologists, primary care providers, mental health professionals, and patient advocates - is critical for holistic care. Education and awareness campaigns can reduce stigma, promote early diagnosis, and empower patients to participate in shared decision-making. Addressing these systemic challenges is essential to unlock the full potential of scientific discovery.

## 13 International collaboration and data sharing

Accelerating progress in PCOS research requires robust international collaboration and open data sharing. Large-scale, multi-center studies and consortia - such as the International PCOS Network - enable the pooling of diverse patient cohorts, harmonization of diagnostic criteria, and validation of novel biomarkers across populations (37).

Adoption of FAIR (Findable, Accessible, Interoperable, Reusable) data principles facilitates integration of clinical, genetic, and real-world datasets, supporting the development of AI-driven models and precision medicine approaches (38). Collaborative initiatives enhance generalizability, reduce duplication, and promote rapid dissemination of best practices. Future efforts should prioritize equitable access to research resources, transparent governance, and meaningful patient engagement in study design and data stewardship.

## 14 A vision for the next decade

The next decade holds promise for a transformative shift in PCOS care - from reactive symptom management to proactive, individualized prevention and treatment. Early diagnosis using non-invasive biomarkers, such as serum AMH, advanced ultrasound imaging, and multi-omics signatures, will enable timely intervention and reduce long-term complications (39).

AI-driven patient stratification may facilitate precision therapies tailored to molecular and phenotypic subtypes. Integrated management approaches addressing reproductive, metabolic, and mental health needs could become standard, supported by multi-disciplinary teams and digital health platforms. Advances in genomics, microbiome science, and wearable technologies will further enhance risk prediction, monitoring, and patient engagement. International collaboration and open data sharing will accelerate validation of new biomarkers and therapies, ensuring equitable access and rapid dissemination of best practices.

By prioritizing patient-centered outcomes, quality of life, and long-term health, the field can realize a future where PCOS is no longer a barrier to well-being and opportunity (40).

## 15 Conclusions

PCOS sits at the intersection of significant scientific opportunity and persistent clinical unmet need. Advances in genomics, neuroendocrinology, and computational medicine have created the conditions for a transition from symptom-based management to biologically targeted, precision therapies. Realizing this potential requires confronting longstanding barriers, including fragmented care pathways, limited biomarker integration, and insufficient alignment between patient priorities and clinical endpoints. Integrating multi-omics profiling, AI-driven stratification, and next-generation biologics represents a transformative path forward. Coordinated action across research, clinical practice, regulatory frameworks, and patient advocacy is essential to close the innovation gap and deliver meaningful, disease-modifying treatments for the millions affected by PCOS.

Achieving this vision will require sustained investment, coordinated global collaboration, and a commitment to integrating biological, computational, and patient-centered innovation across all levels of PCOS research and care.

## Data Availability

The original contributions presented in the study are included in the article/supplementary material. Further inquiries can be directed to the corresponding author.

## References

[1] World Health Organization . (2023). WHO guideline for the prevention, diagnosis and management of infertility. Geneva: WHO. Available online at: https://www.who.int/publications/i/item/9789240115774.

[2] Teede HJ Tay CT Laven J Dokras A Moran LJ Piltonen TT . Recommendations from the 2023 International Evidence‑based Guideline for the Assessment and Management of Polycystic Ovary Syndrome. Hum Reprod. (2023) 38:1655–79. 10.1093/humrep/dead156 PMC10477934 37580037

[3] Azziz R Carmina E Chen Z Dewailly D Laven JSE Legro RS . Polycystic ovary syndrome. (2016) 2:16057. doi: 10.1038/nrdp.2016.57, PMID: 27510637

[4] Lizneva D Suturina L Walker W Brakta S Gavrilova‑Jordan L Azziz R . Criteria, prevalence, and phenotypes of PCOS. (2016) 106:615. doi: 10.1016/j.fertnstert.2016.05.003, PMID: 27233760

[5] Sathyapalan T Atkin SL . Recent advances in PCOS. (2023) 44:378403. doi: 10.1210/endrev/bnad006, PMID: 36879384 PMC10335166

[6] Dewailly D Andersen CY Balen A Broekmans F Dilaver N Fanchin R . Physiology and clinical utility of AMH. (2014) 20:370385. doi: 10.1093/humupd/dmt062, PMID: 24430863

[7] EscobarMorreale HF . Polycystic ovary syndrome: definition, aetiology, diagnosis and treatment. (2018) 14:270284. doi: 10.1038/nrendo.2018.24, PMID: 29569621

[8] Christ JP Cedars MI . Current guidelines for diagnosing PCOS. (2023) 13:1113. doi: 10.3390/diagnostics13061113, PMID: 36980421 PMC10047373

[9] Mohammad Sadeghi H Adeli I Calina D Docea AO Tsarouhas K Buga AM . Polycystic ovary syndrome: pathogenesis and management. (2022) 23:583. doi: 10.3390/ijms23020583, PMID: 35054768 PMC8775814

[10] Dason ES Koshkina O Chan C Sobel M . Diagnosis and management of PCOS. (2024) 196:E85E94. doi: 10.1503/cmaj.230456, PMID: 38286488 PMC10833093

[11] Dunaif A . Insulin resistance and the polycystic ovary syndrome. (2016) 37:487525. doi: 10.1210/er.2015-1104, PMID: 27459230 PMC5045492

[12] Tata B Mimouni NEH Houmard J Thienpont B Clément K Dewailly D . Elevated prenatal AMH programs PCOS in adulthood. (2018) 24:834846. doi: 10.1038/s41591-018-0035-5, PMID: 29760445 PMC6098696

[13] Fitz V Graca S Mahalingaiah S Dokras A Legro RS Teede HJ . Anti-Müllerian hormone neutralization as a therapeutic strategy. (2024) 31:455468. doi: 10.1016/j.tem.2020.02.004, PMID: 33086077

[14] Walters KA Handelsman DJ . Role of steroidogenesis inhibitors in PCOS. (2018) 39:489512. doi: 10.1210/er.2018-00012, PMID: 41613300

[15] Ehrmann DA . Polycystic ovary syndrome. N Engl J Med (2005) 352:1223–1236. doi: 10.1056/NEJMra041536, PMID: 15788499

[16] Skorupskaite K George JT Anderson RA . The kisspeptin–GnRH pathway in human reproductive health and disease. Hum Reprod Update (2014) 20:485–500. doi: 10.1093/humupd/dmu009, PMID: 24615662 PMC4063702

[17] George JT Kakkar R Marshall J Scott ML Finkelstein JS Anderson RA . Neurokinin 3 receptor antagonism decreases gonadotropin and testosterone levels in healthy men. J Clin Endocrinol Metab (2016) 101:4495–4504. doi: 10.1210/jc.2016-2586, PMID: 27571186 PMC5095243

[18] Jensterle M Kravos NA Pfeifer M Kocjan T Janež A Prezelj J . Short‑term intervention with liraglutide improves cardiometabolic risk factors in obese women with PCOS. Eur J Endocrinol (2014) 170:451–459. doi: 10.1530/EJE-13-0890, PMID: 24362411 PMC3922503

[19] Elkind-Hirsch KE Chappell N Shaler D Storment J Bellanger D O’Brien E . A randomized pilot study of empagliflozin for weight reduction in women with PCOS. J Clin Endocrinol Metab (2021) 106:e3937–e3948. doi: 10.1210/clinem/dgab343, PMID: 34000025

[20] Unfer V Facchinetti F Orrù B Giordani B Nestler JE Bizzarri M . Myoinositol effects in women with PCOS: a systematic review of randomized controlled trials. Gynecol Endocrinol (2017) 33:693–699. doi: 10.1080/09513590.2017.1324173, PMID: 41799851

[21] Fitz V Graca S Mahalingaiah S Dokras A Legro RS Teede HJ . Inositol meta-analysis for PCOS. (2024) 109:16301655. doi: 10.1210/clinem/dgad123, PMID: 36869709

[22] Li R Zhang Q Wang Y Chen X Liu J Zhao L . Machine learning for PCOS diagnosis using EHR data. (2022) 130:104081. doi: 10.1016/j.jbi.2022.104081, PMID: 35525400 PMC9674105

[23] Zhao L Xu H Wang S Li Y Chen J Zhang M . Deep learning for ovarian ultrasound interpretation. (2023) 49:345356. doi: 10.1016/j.ultrasmedbio.2022.10.012, PMID: 36543617 PMC10065087

[24] Dapas M Lin FTJ Nadkarni GN Sisk R Legro RS Urbanek M . AI-driven clustering reveals PCOS subtypes. (2023) 21:45. doi: 10.1371/journal.pmed.1003132, PMID: 32574161 PMC7310679

[25] Wang Y Chen X Liu Z Li R Zhang Q Zhao L . Predictive modeling for PCOS risk using AI. (2023) 155:106667. doi: 10.1016/j.compbiomed.2023.106667, PMID: 36805224

[26] Liu J Li X Luo X Zhang Y Chen H Wang S . AI in reproductive endocrinology. (2025) 16:1456. doi: 10.1186/s12958-023-01012-4, PMID: 41853737

[27] Barrera C Torres J Ramirez P Delgado M Alvarez S Rojas L . Machine learning for PCOS phenotyping. (2023) 27:18901902. doi: 10.1109/JBHI.2023.3245678, PMID: 41116384

[28] De Oliveira Trigo L Santos M Pereira R Almeida J Costa L Rodrigues F . AI-enabled reproductive diagnostics. (2025) 31:5572. doi: 10.1093/humupd/dmad112, PMID: 27702463

[29] Morley J Machado CCV Burr C Cowls J Joshi I Taddeo M . Ethical frameworks for AI in medicine. (2025) 31:112120. doi: 10.1016/S2589-7500(20)30113-5, PMID: 41850414

[30] DankwaMullan I . Bias mitigation in AI health systems. (2024) 1:e230021. doi: 10.1126/science.aax2342, PMID: 31649194

[31] Kandarakis E Chatzigeorgiou A Livadas S Palioura E Kandaraki EA Economou F . Endocrine disruptors and PCOS. (2022) 11:e220001. doi: 10.1530/EC-22-0001, PMID: 39659294

[32] Lindheim L Bashir M Munzker J Trummer C Walter M Gmeiner J . Gut microbiome alterations in PCOS. (2017) 12:e0168390. doi: 10.1371/journal.pone.0168390, PMID: 28045919 PMC5207627

[33] Xu N Kwon S Abbott DH Dumesic DA Goodarzi MO Azziz R . Epigenetic mechanisms underlying PCOS. Mol Cell Endocrinol. (2023) 557:111122. doi: 10.1016/j.mce.2022.111122, PMID: 41853590

[34] Torres PJ Siakowska M Banaszewska B Duleba AJ Kelley ST Thackray VG . Gut microbiome and lifestyle interventions in PCOS. (2024) 109:e1234e1245. doi: 10.1210/clinem/dgad012, PMID: 36689278

[35] Jones GL Benes K Clark TL Balen AH Lashen H Ledger WL . The PCOSQ: validation of a quality-of-life questionnaire. (2004) 19:371377. doi: 10.1093/humrep/deh048, PMID: 14747184

[36] Gibson‑Helm M Teede HJ Dunaif A Dokras A Legro RS Moran LJ . Patient-centered approaches in PCOS research and care. (2023) 19:6781. doi: 10.1038/nrendo.2015.197, PMID: 26585657

[37] March WA Moore VM Willson KJ Phillips DIW Davies MJ Norman RJ . The International PCOS Network: advancing research through collaboration. (2022) 37:456464. doi: 10.1093/humrep/deab289, PMID: 35125180

[38] Wilkinson MD Dumontier M Aalbersberg IJ Appleton G Axton M Baak A . The FAIR Guiding Principles for scientific data management. (2016) 3:160018. doi: 10.1038/sdata.2016.18, PMID: 26978244 PMC4792175

[39] Peña AS Witchel SF Boivin J Legro RS Teede HJ Franks S . International evidence-based recommendations for PCOS in adolescents. (2025) 23:151. doi: 10.1038/nrendo.2017.38, PMID: 40069730 PMC11899933

[40] Joham AE Norman RJ Stener‑Victorin E Legro RS Franks S Moran LJ . Polycystic ovary syndrome. Lancet Diabetes Endocrinol. (2022) 10:668–80. 10.1016/S2213-8587(22)00163-2 35934017

